# Supplementary material for: Tumor-associated reactive astrocytes aid the evolution of immunosuppressive environment in glioblastoma
Source: Nat Commun. 2019 Jun 11;10:2541. doi: 10.1038/s41467-019-10493-6 (PMC6559986; doi:10.1038/s41467-019-10493-6)
Supplement: Supplementary file 1 — Supplementary Information [file 41467_2019_10493_MOESM1_ESM.pdf]

Supplementary Figures

Tumor-associated Reactive Astrocytes Aid the Evolution of Immunosuppressive Environment in Glioblastoma

Dieter Henrik Heiland[1,3,11,#], Vidhya M. Ravi[2,3,11,#], Simon P. Behringer[1,3,11], Jan Hendrik Frenking[1,3,11], Julian Wurm[1,3,11], Kevin Joseph[2,3,11], Nicklas W.C. Garrelfs[1,3,11], Jakob Strähle[3,11], Sabrina Heynckes[1,3,11], Jürgen Grauvogel[3,11], Pamela Franco[3,11], Irina Mader[4,5], Matthias Schneider[6], Anna-Laura Potthoff[6], Daniel Delev[7], Ulrich G. Hofmann[2,3,11], Christian Fung[3,11], Jürgen Beck[3,11], Roman Sankowski[8,11], Marco Prinz[8,9,10,11], Oliver Schnell[1,3,11]

[1]*Translational NeuroOncology Research Group, Medical Center, University of Freiburg, Germany*

[2]*Neuroelectronic Systems, Medical Center, University of Freiburg, Germany*

[3]*Department of Neurosurgery, Medical Center, University of Freiburg, Germany*

[4]*Department of Neuroradiology, Medical Center - University of Freiburg, Germany*

[5]*Clinic for Neuropediatrics and Neurorehabilitation, Epilepsy Center for Children and Adolescents, Schön Klinik, Vogtareuth, Germany*

[6]*Department of Neurosurgery, University of Bonn, Bonn, Germany*

[7]*Department of Neurosurgery, University of Aachen, Aachen, Germany*

[8]*Institute of Neuropathology, Medical Center - University of Freiburg,*

[9]*Signalling Research Centres BIOSS and CIBSS, University of Freiburg, Germany*

[10]*Center for NeuroModulation (NeuroModul), University of Freiburg, Freiburg, Germany.*

[11]*Faculty of Medicine, Freiburg University, Germany*

[#] These authors contributed equally

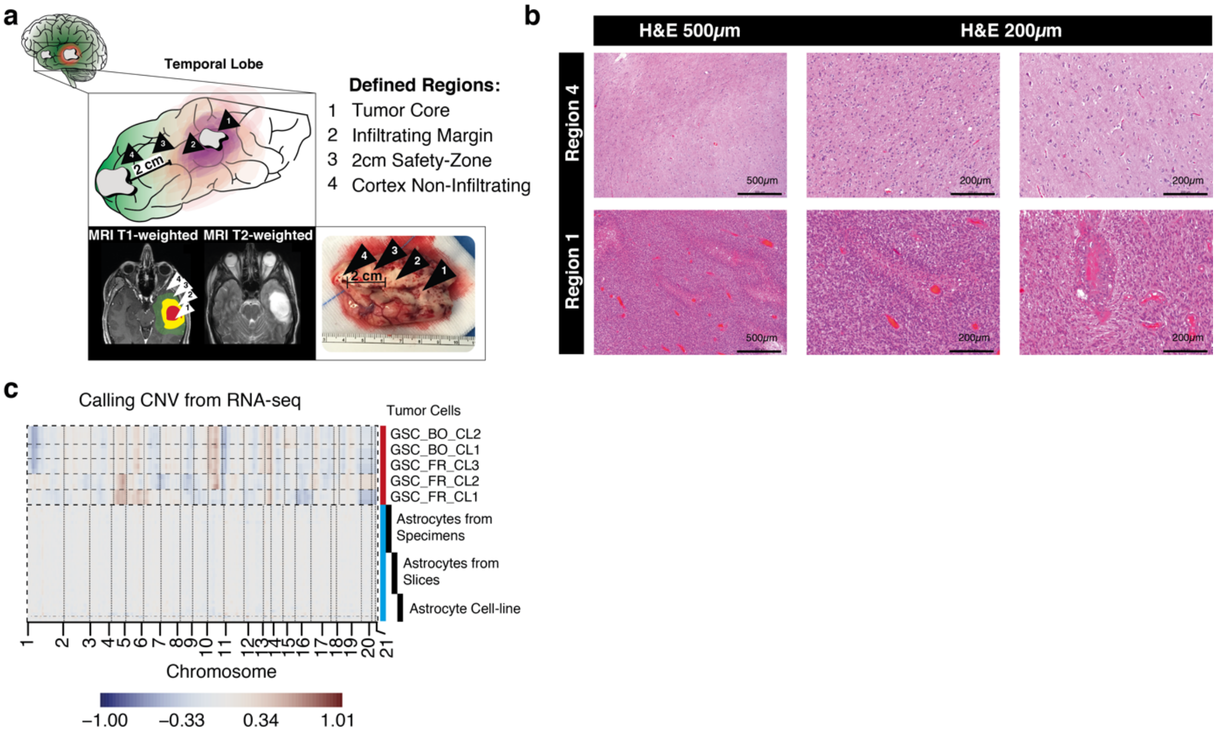

Supplementary Figure1: a) The figure illustrates the preoperative planning to identify the localization for intraoperative sampling. In order to avoid tumor contamination of the “cortex non-infiltrating” region we ensure a minimum distance to MRI signal alteration of at least 2cm (left bottom panel). The tumor was resected on-block and preoperatively planned regions were marked by neuronavigation, controlled in the resected specimen, sampling of the determined regions was done ex-vivo (right bottom panel). To prevent tissue degradation, we performed all sampling steps in the Operation Theater directly. **b)** Representative H&E stainings from region 1 and 4 are illustrated. **c)** In order to validate the tumor cell contamination, we call copy-number-variance of all purified astrocytes and tumor cell lines for control.

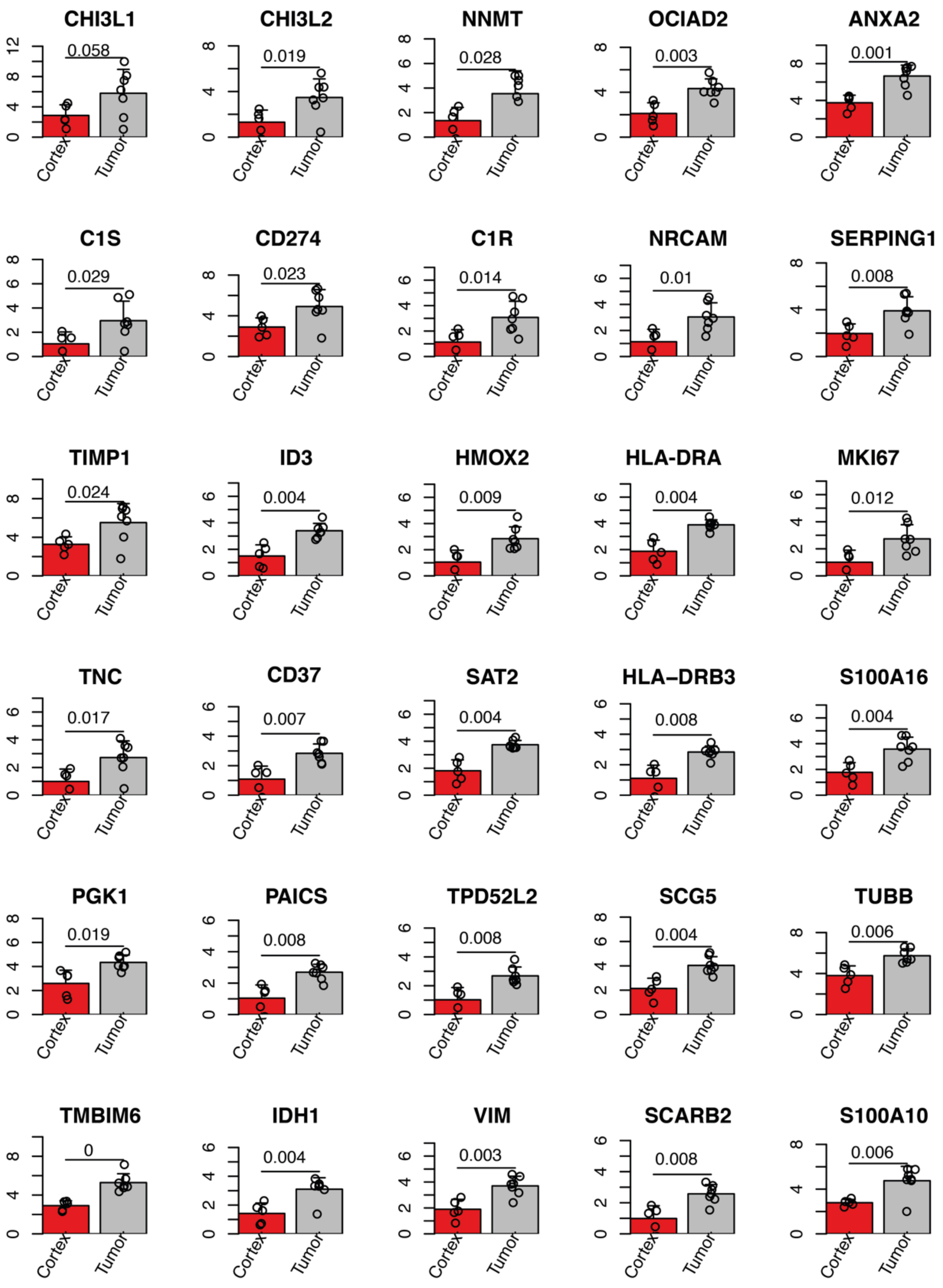

Supplementary Figure2: Bar plots of 30 most up-regulated genes in tumor associated astrocytes vs. astrocytes from non-malignant tissue. Expression values of the x-axis are normalized log2 transformed count data. P-values were determined by two-tailed Student's t-test adjusted by Benjamini-Hochberger for multiple testing. Data is given as mean ± standard deviation.

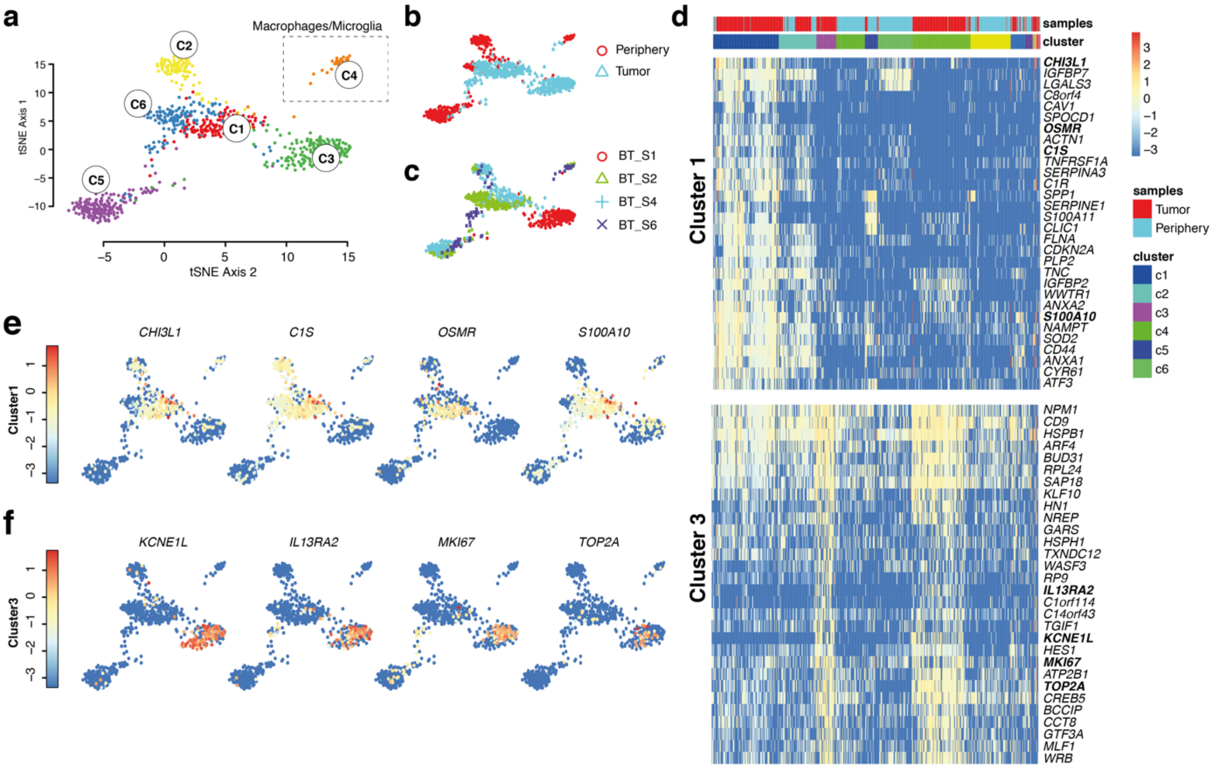

Supplementary Figure3: a) Analysis of single-cell RNA sequencing Data from Darmanis and colleagues. TSNE map of RaceID clustering revealed 5 astrocyte cluster (C4 contained dominantly myeloid cells). With distinct transcriptional differences between tumor and infiltrating regions b). Clustering of most differently expressed genes of the tumor associated genes was illustrated in d) and in colored TSNE maps in e).

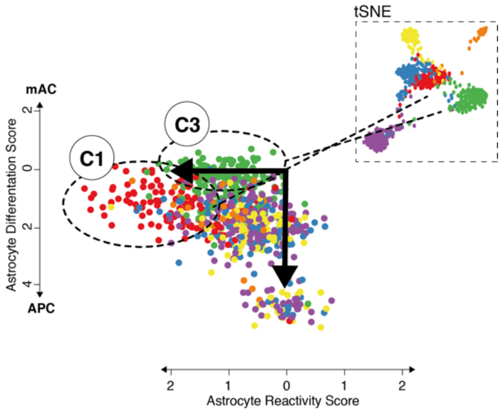

Supplementary Figure4: Two-dimensional scatterplot of astrocytic differentiation and reactivity as shown in Figure 1, from single-cell RNA sequencing Data from Darmanis and colleagues

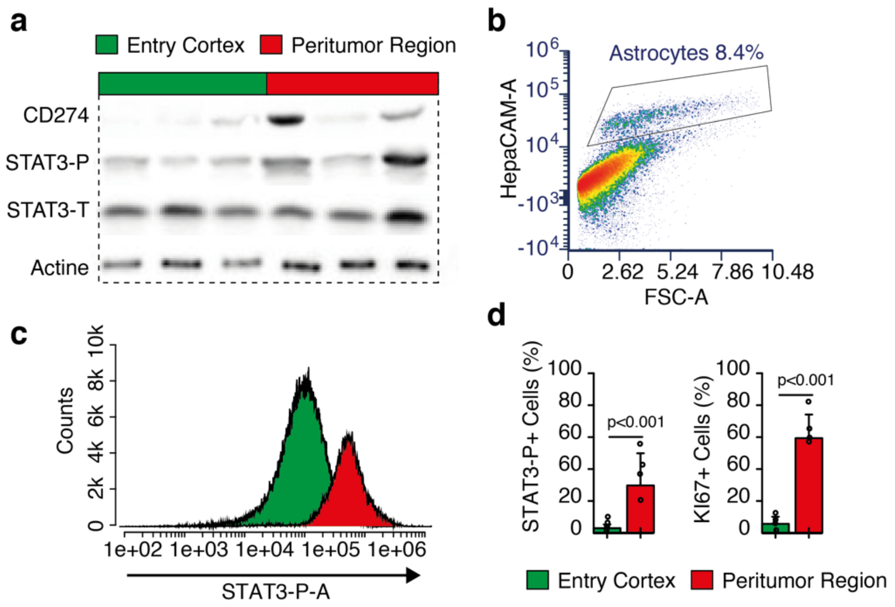

Supplementary Figure5: Workflow und sample collection and Tumor-injection model

a) Western Blot of CD274, phosphorylated (STAT3-P) and total (STAT3-T) STAT3 of purified astrocytes from entry cortex of tumor patients and peritumoral tumor region. Actine is used for loading control **b)** Density plot of FCS area and HepaCAM area from peritumoral tumor region illustrates the gating of astrocytes. Detailed gating strategie is given in the **Supplementary Figure8 c)** Intensity histogram shows the increased amount of STAT3 phosphorylation in peritumoral astrocytes, quantified in the bar plot in **d)**. P-values were determined by two-tailed Student's t-test (d). Data is given as mean ± standard deviation.

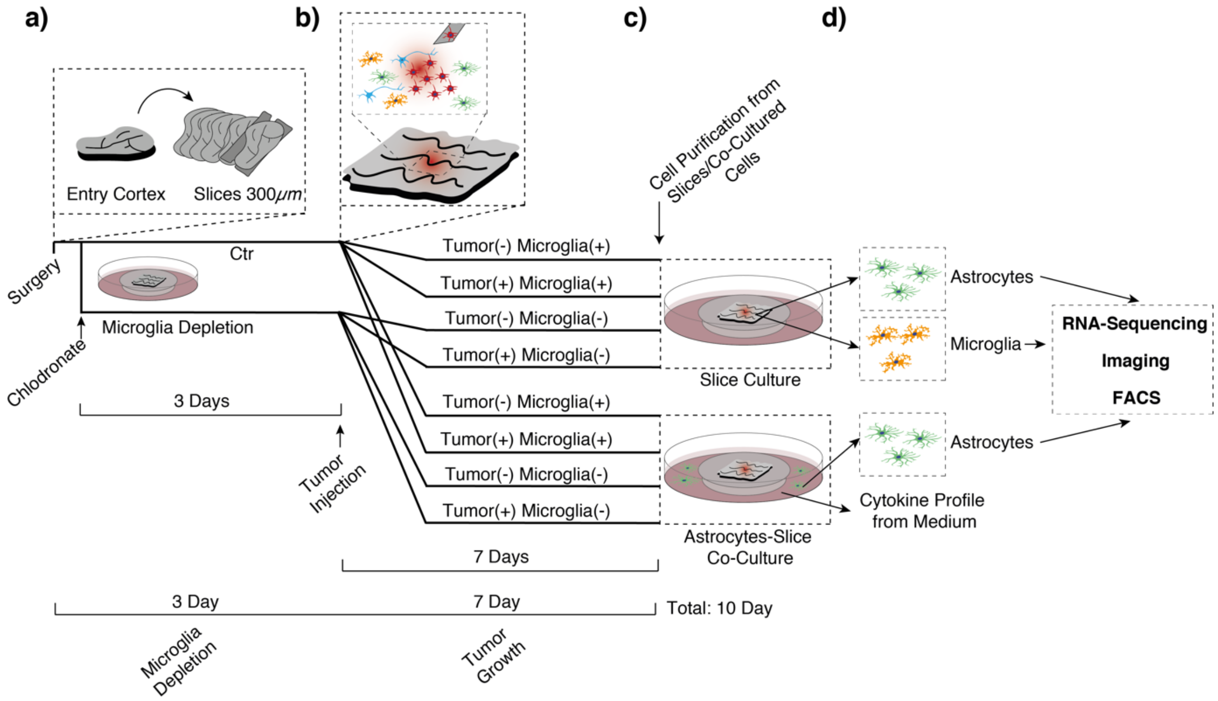

Supplementary Figure6: A detailed illustration is given to show the experimental time course in our novel slice model **a)** After the tumor injection, slices were either used as Microglia(+) or treated with Clodronate (3 days) in order to deplete microglia (Microglia(-) Slices).**b)** After tumor injection slices were incubated for 7 days with and without co-culture of an astrocyte cell line. **c)** After 10 days, astrocytes were purified an analyzed by various methods.

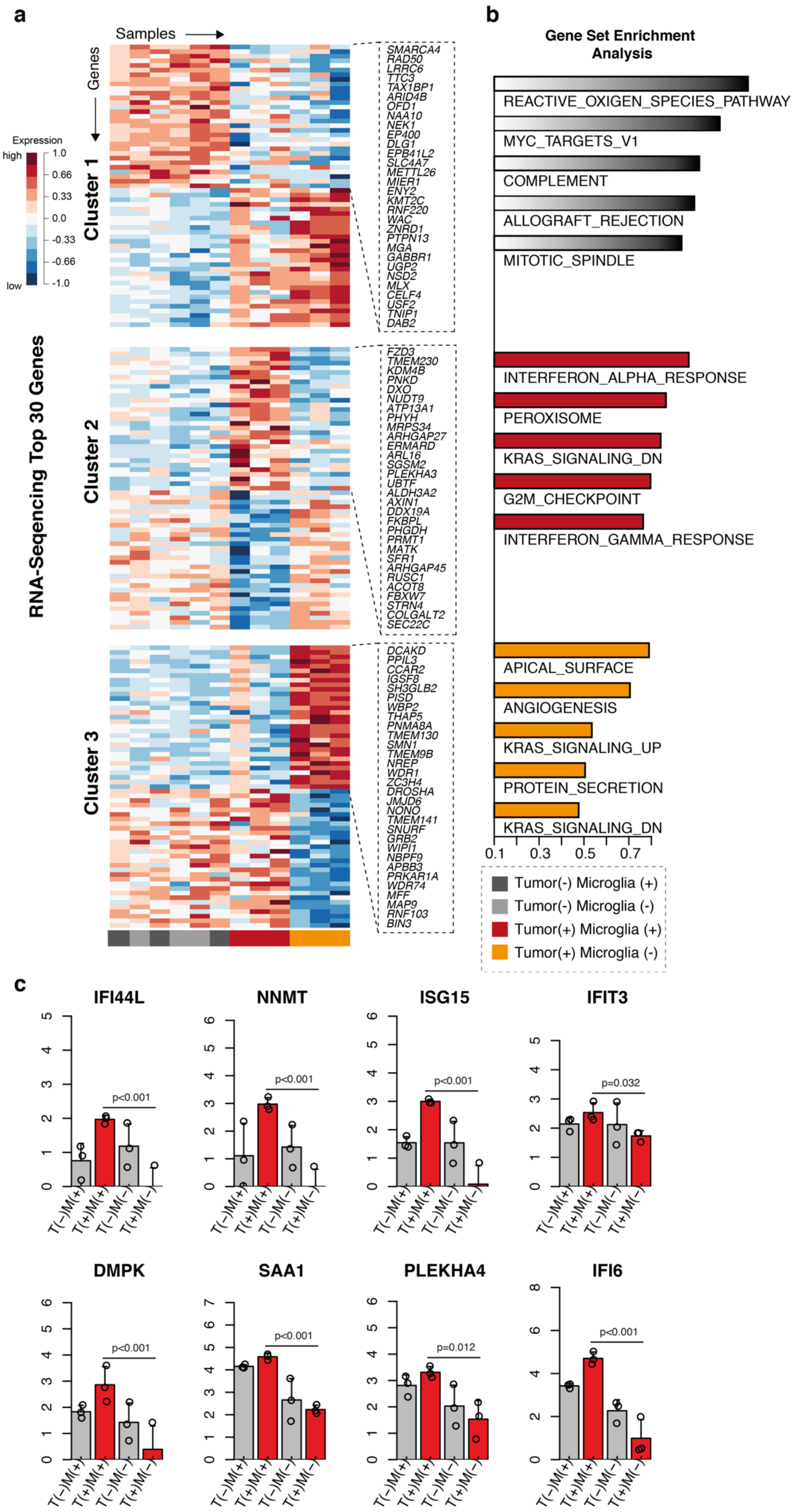

a
Samples
Genes
Expression
high
low
1.0
0.66
0.33
0.0
-0.33
-0.66
-1.0
RNA-Seqencing Top 30 Genes
Cluster 1
Cluster 2
Cluster 3
SMARCA4
RAD50
LRRC6
TTC3
TAX1BP1
ARID4B
OFD1
NAA10
NEK1
EP400
DLG1
EPB41L2
SLC4A7
METTL26
MIER1
ENY2
KMT2C
RNF220
WAC
ZNRD1
PTPN13
MGA
GABBR1
UGP2
NSD2
MLX
CELF4
USF2
TNIP1
DAB2
FZD3
TMEM230
KDM4B
PNKD
DXO
NUDT9
ATP13A1
PHYH
MRPS34
ARHGAP27
ERMARD
ARL16
SGSM2
PLEKHA3
UBTF
ALDH3A2
AXIN1
DDX19A
FKBPL
PHGDH
PRMT1
MATK
SFR1
ARHGAP45
RUSC1
ACOT8
FBXW7
STRN4
COLGALT2
SEC22C
DCAKD
PPIL3
CCAR2
IGSF8
SH3GLB2
PISD
WBP2
THAP5
PNMA8A
TMEM130
SMN1
TMEM9B
NREP
WDR1
ZC3H4
DROSHA
JMJD6
NONO
TMEM141
SNURF
GRB2
WIPI1
NBPF9
APBB3
PRKAR1A
WDR74
MFF
MAP9
RNF103
BIN3
b
Gene Set Enrichment Analysis
REACTIVE_OXIGEN_SPECIES_PATHWAY
MYC_TARGETS_V1
COMPLEMENT
ALLOGRAFT_REJECTION
MITOTIC_SPINDLE
INTERFERON_ALPHA_RESPONSE
PEROXISOME
KRAS_SIGNALING_DN
G2M_CHECKPOINT
INTERFERON_GAMMA_RESPONSE
APICAL_SURFACE
ANGIOGENESIS
KRAS_SIGNALING_UP
PROTEIN_SECRETION
KRAS_SIGNALING_DN
0.1
0.3
0.5
0.7
Tumor(-) Microglia (+)
Tumor(-) Microglia (-)
Tumor(+) Microglia (+)
Tumor(+) Microglia (-)
c
IFI44L
p<0.001
NNMT
p<0.001
ISG15
p<0.001
IFIT3
p=0.032
DMPK
p<0.001
SAA1
p<0.001
PLEKHA4
p=0.012
IFI6
p<0.001
T(−)M(+)
T(+)M(+)
T(−)M(−)
T(+)M(−)

Supplementary Figure7: a) A heatmap of expression data from purified astrocytes of neocortical organotypic slices in four different conditions [Ctr: *Tumor(-) Microglia(+)*, with tumor injection: *Tumor(+) Microglia(+)*, without tumor and microglia depletion: *Tumor(-) Microglia(-)* and tumor injection in microglia depleded slices: *Tumor(+) Microglia(-)*] illustrates an unsupervised clustering by AutoPipe (CRAN: https://cran.rproject.org/web/packages-/AutoPipe/). The clustering revealed 3 different cluster by gap-statistics, cluster composition is given in the bars of the bottom. b) Gene Set Enrichment Analysis of the unique associated genes of each cluster (given by PAMR score), the bar plots illustrate the gene set enrichment for the top 5 pathways. c) Bar plots of 8 most differently regulated genes in ctr tumor associated astrocytes vs. astrocytes from microglia depletion. Expression values of the x-axis are normalized log2 transformed count data. P-values were determined by one-way ANOVA adjusted by Benjamini-Hochberger for multiple testing. Data is given as mean ± standard deviation.

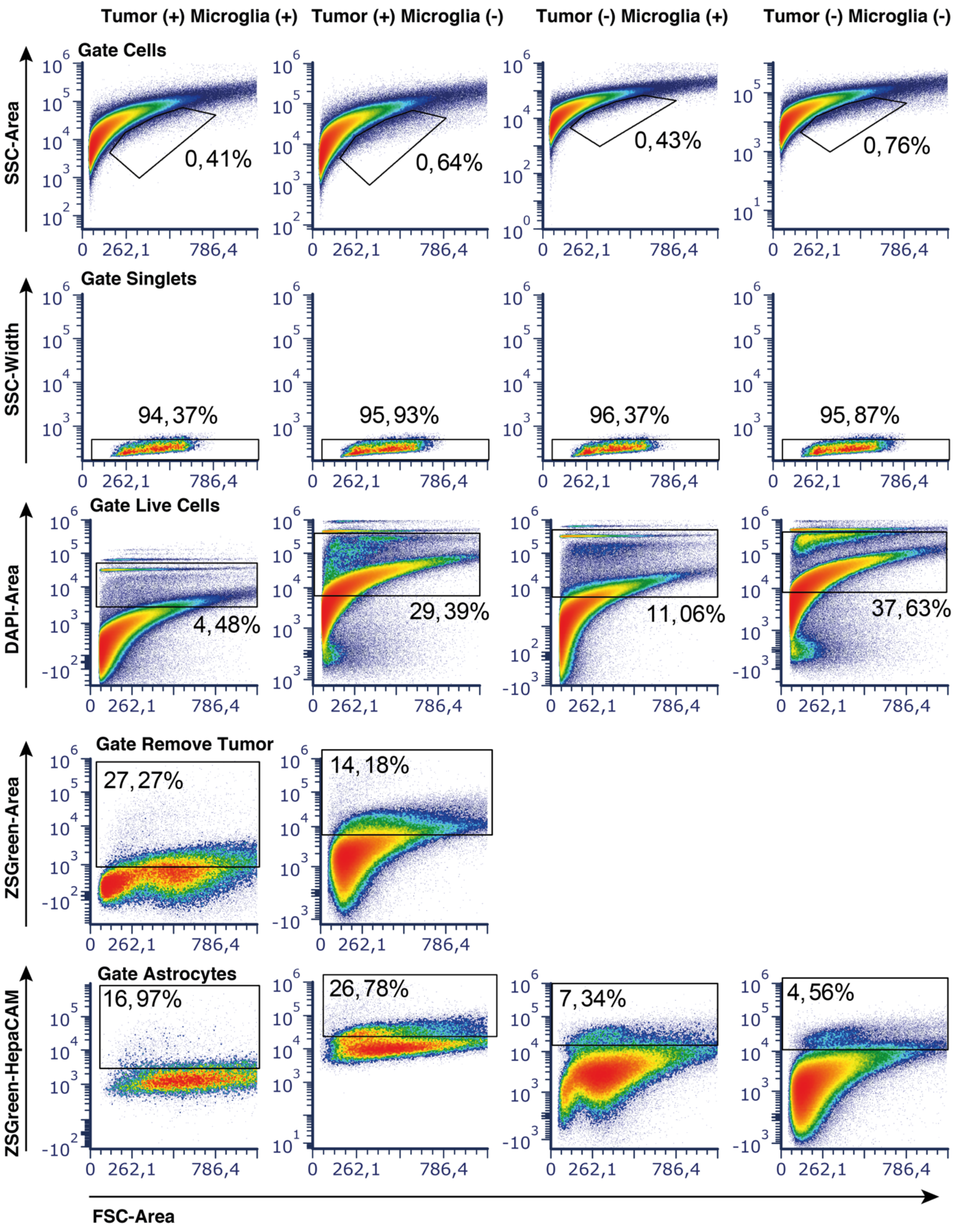

Supplementary Figure8: a) FACS density plot of gating in analysis of our slice model.

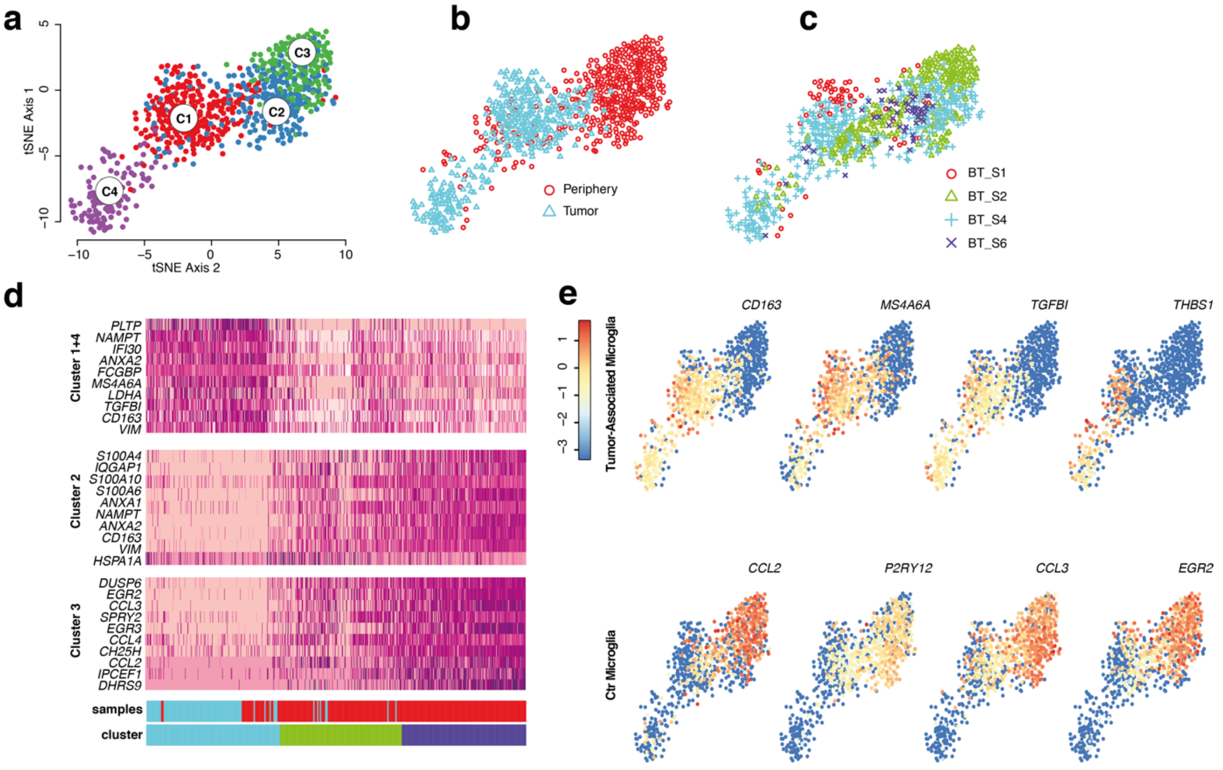

Supplementary Figure9: a) Analysis of single-cell RNA sequencing Data from Darmanis and colleagues. Microglia cells were isolated from the dataset. TSNE map of RaceID clustering revealed 4 microglia cluster with distinct transcriptional differences between tumor and infiltrating regions **b)** and patients **c)**. Clustering of most differently expressed genes of the tumor associated genes was illustrated in **d)** and in colored TSNE maps in **e)**.

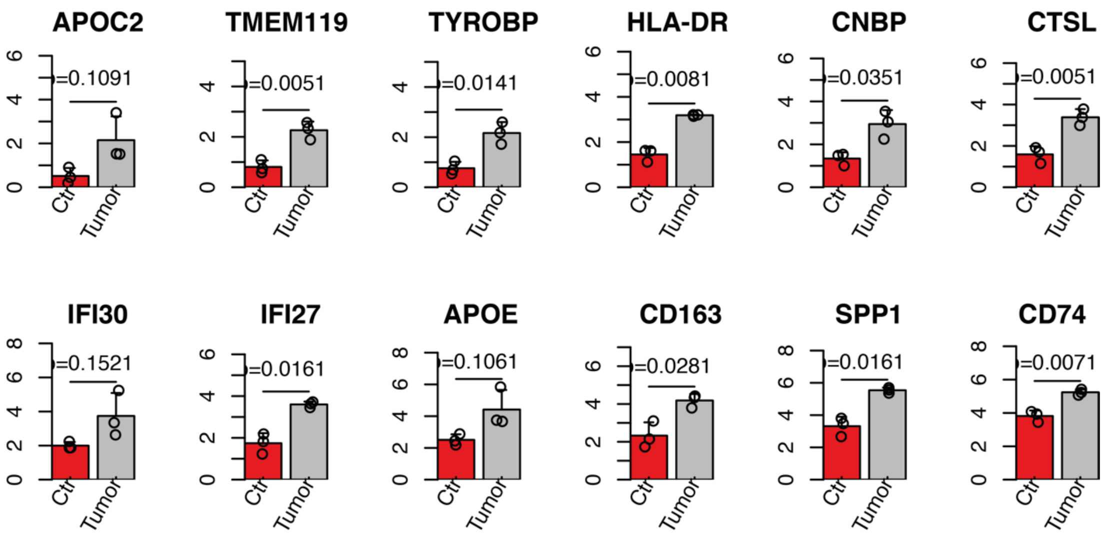

Supplementary Figure10: Bar plots of 12 most up-regulated genes from microglia in tumor injected slices vs. ctr. Expression values of the x-axis are normalized log2 transformed count data. P-values were determined by two-tailed Student's t-test adjusted by Benjamini-Hochberger for multiple testing. Data is given as mean ± standard deviation.

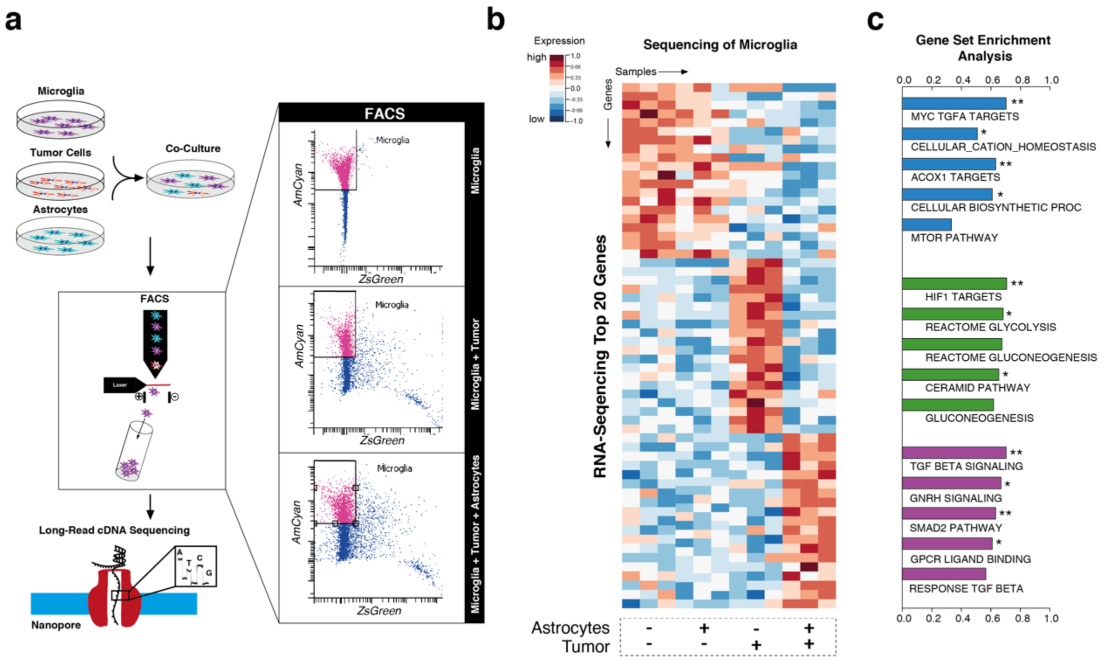

Supplementary Figure11 a) Illustration of the workflow and gating strategy. **b)** AutoPipe unsupervised cluster and heatmap of 20 signature genes of each experimental condition. The panel at the bottom indicates the presence of either microglia tumor cells or astrocytes. **c)** Gene set enrichment analysis (GSEA), highlighting an increased response to TGFß exclusively in the coexistence of astrocytes, tumor cells and microglia. * p-adjusted<0.01, ** p-adjusted<0.001, *** p-adjusted<0.0001.
