# Supplementary material for: Tumor-associated reactive astrocytes aid the evolution of immunosuppressive environment in glioblastoma
Source: Nat Commun. 2019 Jun 11;10:2541. doi: 10.1038/s41467-019-10493-6 (PMC6559986; doi:10.1038/s41467-019-10493-6)
Supplement: Supplementary file 3 — Reporting Summary [file 41467_2019_10493_MOESM3_ESM.pdf]

nature research

Corresponding author(s): Dieter Henrik Heiland

Last updated by author(s): May 5, 2019

Reporting Summary

Nature Research wishes to improve the reproducibility of the work that we publish. This form provides structure for consistency and transparency in reporting. For further information on Nature Research policies, see Authors & Referees and the Editorial Policy Checklist.

Statistics

For all statistical analyses, confirm that the following items are present in the figure legend, table legend, main text, or Methods section.

n/a	Confirmed	
☐	☒	The exact sample size (*n*) for each experimental group/condition, given as a discrete number and unit of measurement
☐	☒	A statement on whether measurements were taken from distinct samples or whether the same sample was measured repeatedly
☐	☒	The statistical test(s) used AND whether they are one- or two-sided<br>*Only common tests should be described solely by name; describe more complex techniques in the Methods section.*
☐	☒	A description of all covariates tested
☐	☒	A description of any assumptions or corrections, such as tests of normality and adjustment for multiple comparisons
☐	☒	A full description of the statistical parameters including central tendency (e.g. means) or other basic estimates (e.g. regression coefficient) AND variation (e.g. standard deviation) or associated estimates of uncertainty (e.g. confidence intervals)
☐	☒	For null hypothesis testing, the test statistic (e.g. *F*, *t*, *r*) with confidence intervals, effect sizes, degrees of freedom and *P* value noted<br>*Give P values as exact values whenever suitable.*
☒	☐	For Bayesian analysis, information on the choice of priors and Markov chain Monte Carlo settings
☐	☒	For hierarchical and complex designs, identification of the appropriate level for tests and full reporting of outcomes
☒	☐	Estimates of effect sizes (e.g. Cohen's *d*, Pearson's *r*), indicating how they were calculated

Our web collection on statistics for biologists contains articles on many of the points above.

Software and code

Policy information about availability of computer code

Data collection	External data were downloaded by GEOquery (3.9).
Data analysis	We used r-software and available packages at Bioconductor and CRAN. Codes are deposited at github.com/Heilandd/ or at the source file. Analysis was mainly performed by a house-build package “AutoPipe”, which is available at CRAN. Detailed Information of the usage of each algorithm is implemented in the method part.

For manuscripts utilizing custom algorithms or software that are central to the research but not yet described in published literature, software must be made available to editors/reviewers. We strongly encourage code deposition in a community repository (e.g. GitHub). See the Nature Research guidelines for submitting code & software for further information.

Data

Policy information about availability of data

All manuscripts must include a data availability statement. This statement should provide the following information, where applicable:

- Accession codes, unique identifiers, or web links for publicly available datasets
- A list of figures that have associated raw data
- A description of any restrictions on data availability

RNA-Sequencing Data available: GSE128536,

Accession codes: www.github.com-/heilandd/.

Further information and requests for resources, raw data and reagents should be directed and will be fulfilled by the Contact: D. H. Heiland, dieter.henrik.heiland@uniklinik-freiburg.de. Full table of all materials is given in the method part.

The source data underlying Figs 1c,e, 2b–c, 3d,f,h, 5e-h and 6c-f are provided as a Source Data file.

Field-specific reporting

Please select the one below that is the best fit for your research. If you are not sure, read the appropriate sections before making your selection.

☒ Life sciences ☐ Behavioural & social sciences ☐ Ecological, evolutionary & environmental sciences

For a reference copy of the document with all sections, see nature.com/documents/nr-reporting-summary-flat.pdf

Life sciences study design

All studies must disclose on these points even when the disclosure is negative.

Sample size	We used 3 biological replications of each experiment. Due to limited resources of human fresh tissue sample size was chosen.
Data exclusions	No data were excluded
Replication	We used 3 biological replications.
Randomization	No randomization
Blinding	We have blinded participants who collect the data on the one hand and who analyze the data on the other hand.

Reporting for specific materials, systems and methods

We require information from authors about some types of materials, experimental systems and methods used in many studies. Here, indicate whether each material, system or method listed is relevant to your study. If you are not sure if a list item applies to your research, read the appropriate section before selecting a response.

Materials & experimental systems

n/a	Involved in the study	
☐	☒	Antibodies
☐	☒	Eukaryotic cell lines
☒	☐	Palaeontology
☒	☐	Animals and other organisms
☐	☒	Human research participants
☒	☐	Clinical data

Methods

n/a	Involved in the study	
☒	☐	ChIP-seq
☐	☒	Flow cytometry
☒	☐	MRI-based neuroimaging

Antibodies

Antibodies used

REAGENTS / RESOURCES SOURCE IDENTIFIER

AffiniPure Goat Anti-Mouse IgG + IgM Jackson ImmunoResearch Laboratories Inc., West Grove, PA, USA 115-005-044
AffiniPure Goat Anti-Rabbit IgG (H+L) Jackson ImmunoResearch Laboratories Inc., West Grove, PA, USA 111-005-003
anti-CD11b (Rabbit) Abcam, Cambridge, UK ab52478
anti-CD45 (Rabbit) Abcam, Cambridge, UK ab10558
anti-CD68 (Mouse) Abcam, Cambridge, UK ab201340
anti-HepaCAM (Human) R&D Systems, Minneapolis, USA MAB4108
Anti-STAT3 (Rabbit) Abcam, Cambridge, UK ab30647
Anti-STAT3-P (Rabbit) Abcam, Cambridge, UK ab76315
Anti-GFAP (Rabbit, donkey) Dako, Santa Clara, USA; Sigma, St. Louis, Missouri, USA Z0334, G9269
Anti-TGFB (Rabbit) Abcam, Cambridge, UK ab92486
Anti- NeuN (Mouse) Millipore, Massachusetts, USA MAB377
Anti- IBA-1 (Rabbit) Wako, Richmond, USA 019-19741
Anti-CD11b (Rabbit) Abcam, Cambridge, UK Ab133357
Anti-α-Tubulin (Mouse) Santa Cruz Biotechnology, Texas, USA Sc-8035
Anti- Ki67 (Rabbit) Abcam, Cambridge, UK Ab15580
DAPI Sigma, Missouri, USA 32670
Goat anti-Mouse IgG Alexa Fluor 488 Life Technologies Coorperation Eugene, USA A11001
Goat anti-Rabbit IgG Alexa Fluor 568 Life Technologies Coorperation Eugene, USA A11011
Donkey anti-Goat IgG Alexa Fluor 647 Life Technologies Coorperation Eugene, USA A21447
Goat anti Rabbit IgG Alexa Fluor 488 Life Technologies Coorperation Eugene, USA A11008

Donkey anti- rabbit IgG Alexa Fluor 555 ThermoFisher Scientific, Massachusetts, USA
Goat anti-Mouse IgG-HRP Santa Cruz Biotechnology, Texas, USA Sc-2005
Goat-anti-Rabbit IgG-HRP Santa Cruz Biotechnology, Texas, USA Sc-2004
Mouse-anti-Goat IgG-HRP Santa Cruz Biotechnology,Texas, USA Sc-2354
PE/CY5 conjugation kit Abcam, Cambridge, UK Ab 102893
APC/CY7 conjugation kit Abcam, Cambridge, UK Ab 102859
Ki-67 efluor 660 (SolA15) ThermoFisher Scientific, Massachusetts, USA 50-5698-82

Validation

All antibodys validated by the manufacturer

Eukaryotic cell lines

Policy information about cell lines

Cell line source(s)

The cell lines used are a primary GBM cell lines (ID#GSC-CL1),(ID#GSC-CL2),(ID#GSC-CL3), University of Freiburg,
Astrocytes: SVG p12 (ATCC® CRL-8621™)
Microglia: (T0251) ABM

Authentication

Authentication was provided by the manufacturer

Mycoplasma contamination

All cell lines are regularly checked for mycoplasma contamination

Commonly misidentified lines
(See ICLAC register)

Not Listed

Human research participants

Policy information about studies involving human research participants

Population characteristics

The samples were selected from patients undergoing surgical resection, selected by their tumor (GBM, IDH-WT).

Recruitment

The samples were selected from patients undergoing surgical resection, special recruitment is not provided in the study.

Ethics oversight

100020/09 and 472/15_160880

Note that full information on the approval of the study protocol must also be provided in the manuscript.

Flow Cytometry

Plots

Confirm that:

☒ The axis labels state the marker and fluorochrome used (e.g. CD4-FITC).

☒ The axis scales are clearly visible. Include numbers along axes only for bottom left plot of group (a 'group' is an analysis of identical markers).

☒ All plots are contour plots with outliers or pseudocolor plots.

☒ A numerical value for number of cells or percentage (with statistics) is provided.

Methodology

Sample preparation

Included in the methods:

For cells: Astrocytes (mCherry) and tumor cells (ZsGreen) or microglia (AmCyan) were sorted by FACS Aria III (BD Bioscience) in the core facility, Medical-Center Freiburg, University of Freiburg in accordance to the manufacturer's instructions.
For slices: Tissue specimens were mechanically dissociated using a glass homogenizer on ice and sequentially passed through 100 µm and 40 µm nylon cell strainers (BD Falcon #352360 and #352340). The mesh was then rinsed several times with 4°C cold PBS/EDTA. Resulting cell suspensions can be kept on ice for up to 20 minutes while other tissue samples are being processed. After centrifugation (310 g; 4°C; 6 min) and removal of the supernatant, the cell pellet was suspended in 0.5 ml 4°C cold PBS/EDTA. Five ml of -20°C cold 80% methanol was added drop-wise under constant, gentle vortexing. Samples were incubated for 30 min on ice and subsequently overnight at -20°C before being subjected to staining. Alternatively, samples can be stored at -20°C for up to 1 year. The cell suspension was washed and centrifuged at 350xg for 5 mins and cells were counted to be roughly 2 x 106. This is further followed by resuspending the cells using permeabilization buffer (0.1% Triton X-100 in 1X PBS) for 5 mins at room temperature. Samples were centrifuged briefly and the pellets were washed 2 times with 1X PBS. 5 µl of TruStain FcXTM were added per million cells in 100 µl staining volume to avoid unspecific antibody binding (It is not necessary to wash the cells between these blocking and immunostaining steps. Cells were stained with fluorochrome-conjugated antibodies). Antibodies were directly conjugated with the following fluorescent tags: PE/Cy5 and APC/Cy7. The following antibodies were used: Anti-

STAT3, HepaCAM, Ki67 efluor 660 and DAPI. Antibody staining was performed according to the manufacture instructions. Finally, cells were washed and resuspended in at least 0.5 to 1 mL of FACS buffer depending on the number of cells.

Instrument	Sony SP6800 spectral analyzer
Software	FCS Express 6 plus
Cell population abundance	Astrocytes (HepaCAM+, ZsGreen(-))
Gating strategy	Gating strategies are present in the supplementary information.

☒ Tick this box to confirm that a figure exemplifying the gating strategy is provided in the Supplementary Information.
